# Screw placement in arthroscopically assisted osteosynthesis of radial head fractures using a reference k-wire in the radiocapitellar joint: a cadaveric study

**DOI:** 10.1007/s00402-022-04605-z

**Published:** 2022-10-05

**Authors:** Roland S. Camenzind, Davide Cucchi, Tim Leschinger, Michael Hackl, Lars P. Müller, Kilian Wegmann

**Affiliations:** 1 Center for Orthopedic and Trauma Surgery, University Hospital Cologne, University of Cologne, Cologne, Germany; 2 Department of Orthopedics, Balgrist University Hospital, University of Zurich, Forchstrasse 340, 8008 Zurich, Switzerland; 3 Department of Orthopedic and Trauma Surgery, Cantonal Hospital Lucerne, Spitalstrasse, 6000 Lucerne, Switzerland; 4 Department of Orthopedic and Trauma Surgery, University Hospital Bonn, Sigmund-Freud-Str. 25, 53127 Bonn, Germany

**Keywords:** Elbow arthroscopy, Arthroscopic-assisted, Radial head fractures, Reference k-wire, Screw osteosynthesis

## Abstract

**Purpose:**

The optimal screw placement in arthroscopically assisted fixation of radial head fractures is still an issue and no guiding methods have been evaluated in the recent literature. The study hypothesis was that using a “reference k-wire” percutaneously inserted in and parallel to the radiocapitellar joint would enable to achieve a trajectory more parallel to the radial head articular surface as compared to a free-hand k-wire placement.

**Methods:**

Arthroscopically assisted placement of a k-wire in the radial head was performed in seven fresh-frozen human cadaver specimens by three surgeons. Three different techniques were evaluated: freehand drilling (technique A), placement using a “reference” k-wire in the radiocapitellar joint as a reference without (technique B), and with the AO parallel k-wire guide (technique C). Radiographs from all procedures were obtained and the inclination angle “*α*” between the k-wire and the articular surface of the radial head was measured and compared among the techniques.

**Results:**

Angles of 84 radiographs were obtained and showed a mean α angle of 30.1° ± 13° for technique A, 5.7° ± 4.5° for technique B, and 5.4° ± 3.7° for technique C. The angle α was significantly higher with technique A as compared to B (*p* < 0.0001) and C (p < 0.0001). There was no difference between methods B and C (n.s.). No difference was observed among the surgeons for all three methods (*p* = 0.66).

**Conclusion:**

With the use of an additional “reference” k-wire placed in the radiocapitellar joint, the guiding k-wire for screw drilling can be placed almost parallel to the radial head joint line with limited variability and a good reproducibility during arthroscopically assisted radial head fracture fixation.

**Clinical relevance:**

The here-presented method of an additional, percutaneous introduced “reference” k-wire is easily applicable and helpful to achieve parallel screw placement during arthroscopically assisted radial head fracture fixation.

**Level of evidence:**

IV, biomechanical cadaver study

## Introduction

The radial head is crucial for the stability and function of the elbow joint [1–3]. For the treatment of radial head fractures arthroscopic-assisted techniques for fixation, in specific fracture types, are gaining popularity and present advantages compared to the classic open approach [4–6]. Arthroscopy offers potential for better visualization of the articular surface and, with it, improved understanding of the fracture morphology. With this opportunity, a more precise anatomical reduction of the articular surface and the simultaneous treatment of concomitant injuries are potentially possible [5].

Different cadaver studies [7, 8] showed a possible exposure of the whole radial head circumference with standard arthroscopic portals for adequate screw placement. Screw placement is the crucial step in fixation of radial head fractures. As known from traumatology principles, increased primary stability of screws is achieved when they are placed perpendicular to the fracture line [9]. Most radial head fractures type I and II according to the Mason classification [10] modified by Hotchkiss [11] are partial intra-articular fractures: in these cases, axial, valgus, and external rotatory loading at the radiocapitellar joint leads to shearing of the radial head resulting in an intra-articular fracture in which in most cases the fracture line is perpendicular to the radial head articular surface, as described by Gordon et al. [12]. Hereby, the perfect screw inclination would be at an angle of 0° to the radial head articular surface.

Positioning the guide wire can be difficult during elbow arthroscopy since only a two-dimensional view of the radial head is provided and determining the radial longitudinal axis is challenging. Malpositioning of the k-wire can lead to increased surgical time, a higher amount of X-ray radiation to patient and surgeon, and at last improper screw placement. The purpose of this study was to evaluate the performance of two guiding methods and compare them to a free-hand technique of k-wire placement in a cadaveric elbow arthroscopy situation. The study hypothesis was that using a “reference k-wire” inserted in the radiocapitellar joint as a reference for “guide k-wire” placement would enable to achieve a trajectory more parallel to the radial head articular surface as compared to a free-hand k-wire placement. The possible role of an AO parallel k-wire guide in assisting k-wire placement was also evaluated.

## Materials and methods

This cadaver study was approved by the institutional review board (21-1118). For the present study, seven fresh-frozen cadaver specimens of upper extremities from human donors were used (females: 57%; right elbows: 57%; median age at death: 76 years). The specimens were evaluated for visible or radiological signs of previous trauma or deformity.

Three treatment groups were defined for subsequent comparison (techniques A, B, and C). Technique A consisted in free-hand positioning of a 2.0 mm guide k-wire in the radial head under sole visual arthroscopic control. For technique B, firstly, a “reference k-wire” was positioned percutaneously in the radiocapitellar joint and used as a reference to place the guide k-wire in the radial head. In technique C the “reference k-wire” was positioned percutaneously in the radiocapitellar joint (in the same way as in technique B) and, additionally, the AO parallel k-wire guide was used to drill the guide k-wire in the radial head.

All seven obtained specimens were allocated in a random sequence to each of the three treatment groups so that a sequence of 21 procedures was generated before the study begin. All three procedures were performed in each of the seven specimens.

**Fig. 1:**
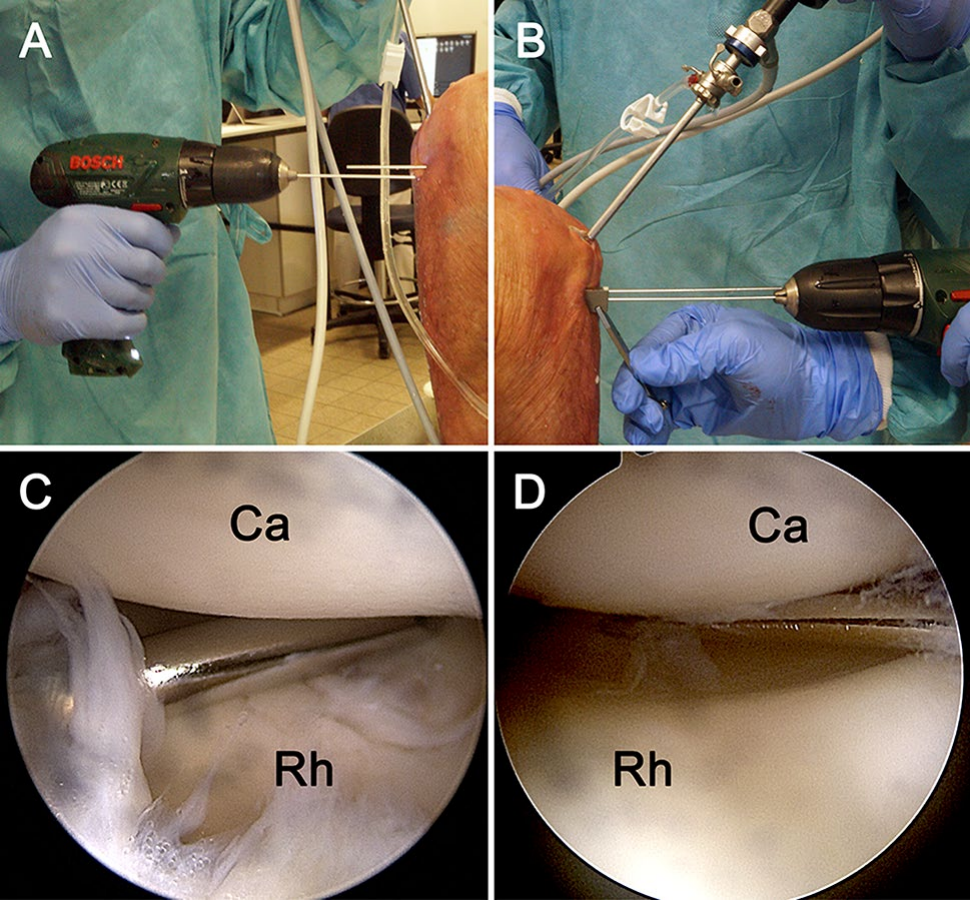
Drilling the guiding k-wire into the radial head without (**A**) and with an AO parallel k-wire guide (**B**). Arthroscopic view of the radiocapitellar joint (*Ca* capitellum; *Rh* radial head) through a high posterolateral portal (left **A**, right **B**) while placing a “reference” k-wire into the radiocapitellar joint from the lateral soft-spot portal

Standard arthroscopy (Synergy, Arthrex, Naples, FL, USA) was performed with the elbow positioned at 90° of flexion, imitating patient positioning in lateral decubitus (Fig. 1). A high posterolateral portal was established for diagnostic arthroscopy. If necessary, an intra-articular debridement with a shaver was performed for better visualization via a standard anterolateral portal. After a single trial of positioning of the guide k-wire with the desired technique, an x-ray with a strict lateral view of the radial head and in the plane of the inserted guide k-wire was obtained with digital fluoroscopy (Fluoroscan Insight-FD Mini, Hologic Medicor GmbH, Kerpen, Germany).

After being instructed on the treatment groups and procedures, three orthopedic surgeons (RSC, DC, KW) performed each of the three techniques on the seven specimens in one sequence of 21 arthroscopies: these procedures were evaluated to assess inter-rater reliability; furthermore, to assess intra-rater reliability of the procedure, the most experienced of the three surgeons (KW) performed a second sequence of 21 arthroscopies 4 h after the first sequence. To avoid repeated perforations of the radial head at the same k-wire insertion point, the elbow was fixed in different pronation and supination positions.

**Fig. 2:**
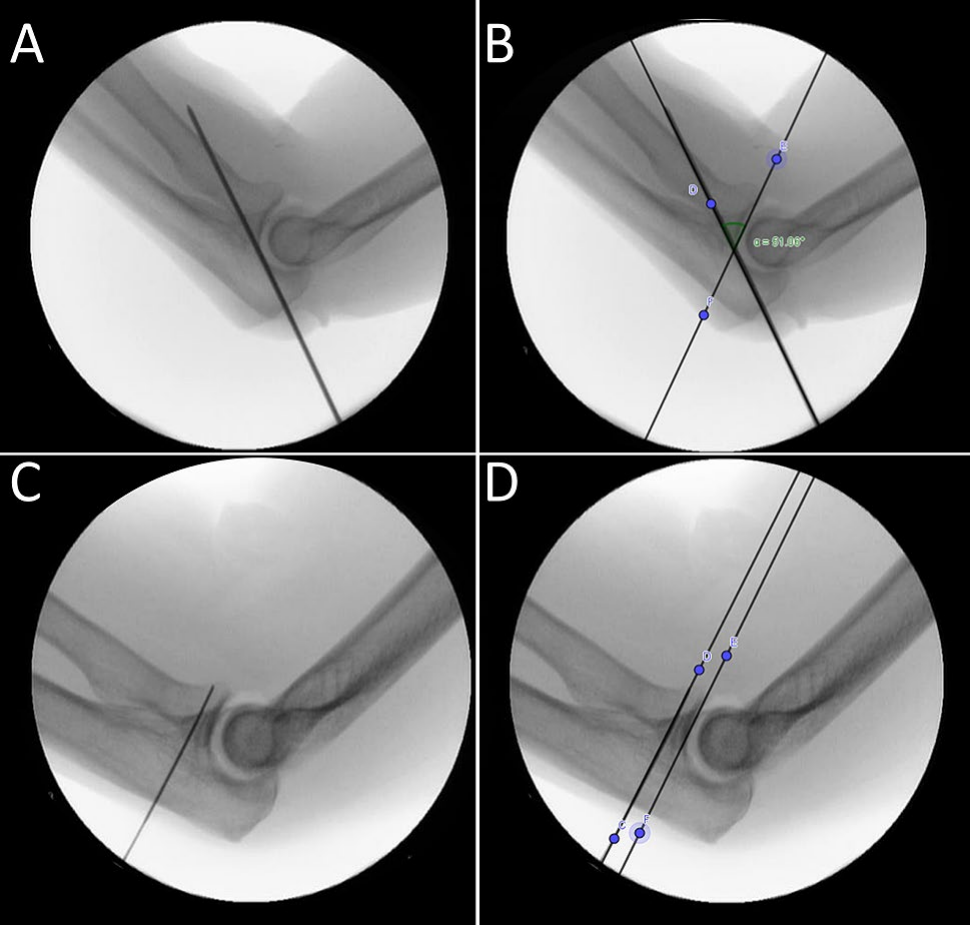
Radiographs after placement of the guiding k-wire with the corresponding measurement of the inclination angle α. Radiograph after free-hand placement of the guiding k-wire in the radial head (**A**) showing an inclination angle of 51° (**B**), and radiograph after using a reference k-wire in the radiocapitellar joint without parallel k-wire guide (**C**) and an almost parallel guide k-wire in the radial head (**D**)

The inclination angle “α” between the guide k-wire and the articular surface of the radial head was determined on digital radiographs with two decimals accuracy using dedicated software (GeoGebra 6.0.620, Hohenwarter et al., http://www.geogebra.org, 2020) (Fig. 2).

Two examiners blinded to the group allocation of the radiographs (RSC, DC) analyzed all radiographs independently. Before the study, both observers were instructed about the measurement techniques. Two weeks after the initial measurement, one surgeon (RSC) re-measured all inclination angle α to evaluate the intra-observer reliability of the radiographic measurement.

### Statistical analysis

Based on the previously published data concerning screw placement in radial head fractures [7], an a priori power analysis was calculated to detect a significant difference between two different angles using the freely available software G*Power 3 (Erdfelder, Faul, Buchner, Lang, HHU Düsseldorf, Düsseldorf, Germany). A sample size of at least 6 elbows with an *α* = 0.05 for a power of 95% was established to detect a change between two angles (two-sided *t*-test for two different groups).

Reliability was evaluated using intraclass correlation coefficients (ICCs) and a two-way mixed-effect model assuming a single measurement and absolute agreement.

Calculated means from both readers were then used to analyze and present data. Normal distribution of the data was tested with a Kolmogorov–Smirnov test. Parametric (paired *t*-test) or non-parametric *t*-tests (Wilcoxon matched-pairs signed-rank test) were applied to compare the different conditions. To evaluate the differences among the methods one-way analysis of variance (ANOVA) was performed.

The significance level was set at 0.05 and the results are reported as means with standard deviation if not stated otherwise. The statistical analyses were computed using MedCalc Statistical Software version 19.6 (MedCalc Software bv, Ostend, Belgium; https://www.medcalc.org; 2020).

## Results

Angles of 84 radiographs were obtained and analyzed. Inter- and intra-observer reliability of the measurement of the inclination angle α measured with ICC was 0.9855 (95% CI 0.9774–0.9906) and 0.9834 (95% CI 0.9673–0.9907) respectively.

**Fig. 3:**
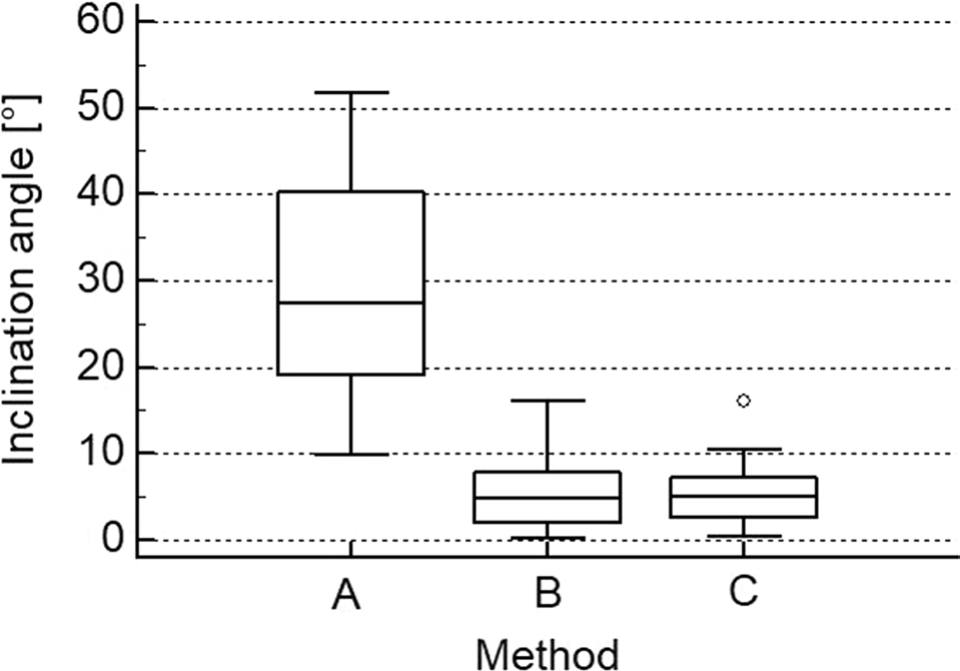
Boxplot of the inclination angle α with the three techniques: **A** free-hand drilling, **B** using a “reference” k-wire in the radiocapitellar joint, and **C** additional AO parallel k-wire guide for the drilling of the guide k-wire

With the technique A, a mean *α* angle of 30.1° ± SD 13 was obtained by the three surgeons; with the technique B the mean α angle dropped to 5.7° ± SD 4.5° and with the technique C to 5.4° ± 3.7 (Fig. 3). A significant difference was observed when comparing technique A to both technique B (*p* < 0.0001) and C (*p* < 0.0001). There was no difference between techniques B and C (*p*: n.s.). No difference was observed among the three surgeons (n.s.), neither for technique A (n.s.), B (n.s.) or C (n.s.). Inter-observer reliability measured with ICC for method A was 0.6097 (95% CI − 0.07 to 0.92), method B 0.7319 (95% CI 0.08–0.95) and method C 0.7652 (0.29–0.95). Intra-observer reliability for the second sequence of the senior author (KW) showed an ICC of 0.7781 (95% CI 0.46–0.91).

No damage to the radiocapitellar cartilage was observed arthroscopically during the study period.

## Discussion

The main finding of this study is that the use of an additional “reference” k-wire in the radiocapitellar joint can support to drilling the definitive guide k-wire for the cannulated screw with less variability and almost parallel to the radial head joint line with good reproducibility during arthroscopic-assisted radial head fracture fixation. A high agreement between surgeons can be achieved either by using the k-wire as a simple reference or with the additional use of the AO parallel k-wire guide.

Arthroscopy of acute elbow trauma is becoming more and more popular and has its role in the fixation of radial head fractures [13]. Arthroscopy allows direct visualization of the fracture and with this perfect reduction of this articular fracture can be verified [14]. Further advantages are reduced surgical trauma to the soft tissue compared to an open approach, the possibility to address other intra-articular soft-tissue lesions, and evacuation of loose bodies in the elbow joint. Clinical results of arthroscopic-assisted osteosynthesis are encouraging [6, 15, 16].

The techniques presented here can help surgeons place the guide wires for cannulated screw in a more parallel trajectory to the radial head articular surface (inclination angle α tend towards zero), herewith potentially enhancing the biomechanical performance of fixation and facilitating arthroscopic-assisted fracture management of radial head fractures. Surgical treatment of radial head fracture is indicated in some Mason type II, type III, and IV fractures while only Mason type II fractures so far allow an arthroscopic-assisted approach. Two-part fractures with the fracture fragment still close to the radial head articular surface are ideal candidates for arthroscopic surgery. Fragments which are dislocated outside the radiocapitellar joint usually are not amenable to being addressed by arthroscopic techniques [17].

In a cadaver study analyzing the inclination angle α of a k-wire introduced by different arthroscopic standard portals, Cucchi et al. [18] showed a mean α angle ranging from 14° to 25° obtained from the medial, 17° from the anterolateral, and − 2.6 for the more distal portal. The authors concluded a more parallel trajectory of the screw about the radial head surface is desirable to achieve superior biomechanical stability. With the here-presented additional “reference k-wire” in the radiocapitellar joint, an α angle very close to 0° (5.7° without and 5.4° with the AO parallel k-wire guide) could be achieved. Notably, the standard deviation documented in the present study using the “reference k-wire” was markedly lower (less than 5°) than the standard deviation reported both without the “reference k-wire” and that documented in the previously cited study (both 13°), suggesting a reduced variability of k-wire placement with the newly proposed technique. The application of this additional k-wire is easy, time-saving, cheap and even for inexperienced arthroscopists easily applicable. Through the soft-spot portal, or wherever the fracture line demands its placement, the k-wire can be introduced and pushed forward to the anterior elbow joint. The correct position of this wire can be assessed arthroscopically and the direction of the wire macroscopically. Care should be taken not to advance this sharp instrument too far anteriorly to avoid damage to the neurovascular structures. Moreover, the surgeon must take care, that the k-wire is not bent by the soft tissues, which would compromise the guiding effect. Further, excessive distalization of the lateral portal should be avoided and pronation of the forearm while cutting/screwing from the lateral is warranted to displace the posterior interosseous nerve medially and thus increase safety.

The high variety and the steep inclination angle obtained with the free-hand k-wire technique underline the necessity for an improved technique. Several factors add to the difficulty of free-hand k-wire placement, like soft-tissue entanglement around the k-wire, blurred vision by fracture hematoma, and resistance to placement by the annular ligament. The annular ligament reaches up to the articular surface of the radial head and is tightly fit around the radial head. To be able to place the k-wire at the correct depth to avoid intra-articular placement, the guiding k-wire must be placed at about 6–8 mm below the radial head surface. Therewith, its entry point can be blocked by the annular ligament. In a conventional technique, the k-wire needs to pull down the annular ligament to gain visibility on the entry point. In the author’s perception, this step mainly adds to misplacement and variability in the α angle. With the described techniques B and C, this step is avoided, as the “reference” k-wire dictates the direction of the guiding k-wire, which can thus be easily placed also through the annular ligament.

### Limitations

There are several limitations of this study. First, this is an anatomic cadaver study and all portals were placed by a single, experienced surgeon. In a real surgical setup, the position of the standard portals may differ based on the surgeons’ preferences. Furthermore, the experimental setup did not include the fracture reduction into account. Arthroscopic management in an acute trauma case can be much more difficult and is highly demanding and advanced skills in elbow arthroscopy are needed. Third, there is a high risk of bias by repeated use of the different specimens, and perforating the radial head several times with a 2.0 mm k-wire. To avoid introducing the k-wire in the previous perforation, the elbow was fixed in different pronated and supinated positions. Fourth, the parallelism of osteosynthetic screws to the radial head articular surface is not known to correlate with superior clinical outcomes, nor is this stated by the authors. The authors merely proclaim known biomechanical basics, which offer improved primary stability if screws are placed perpendicular to the fracture line. But even more, the present paper does not focus on the potential superiority of a specific k-wire position but wants to display the feasibility of a simple technique for navigated k-wire placement. This study shows within its limitation that the definitive screw placement can be navigated using simple tools.

## Conclusion

With the use of an additional “reference” k-wire placed in and parallel to the radiocapitellar joint, the k-wire for consecutive screw drilling can be navigated, resulting in an almost parallel position about the radial head joint line, with good reproducibility during arthroscopically assisted radial head fracture fixation.
